# Hematological Toxicity During Concomitant Treatment With Ruxolitinib and Avelumab for Merkel Cell Carcinoma

**DOI:** 10.3389/fonc.2020.579914

**Published:** 2020-10-22

**Authors:** Luciana Buonerba, Rossella Di Trolio, Antonio Grimaldi, Aniello Tucci, Emilio Leo, Concetta Ingenito, Ferdinando Costabile, Gianluca Ragone, Beatrice Savastano, Maria Teresa Uzzauto, Valeria Belsito Petrizzi, Giuseppe Di Lorenzo

**Affiliations:** ^1^Oncology Unit, “Andrea Tortora” Hospital, Azienda Sanitaria Locale Salerno, Pagani, Italy; ^2^Unit of Melanoma, Cancer Immunotherapy and Development Therapeutics, Istituto Nazionale Tumori Istituto di Ricovero e Cura a Carattere Scientifico Fondazione G. Pascale, Naples, Italy; ^3^Oncology Unit, “Santa Maria La Pietà” Hospital, Azienda Sanitaria Locale Napoli 3 Sud, Nola, Italy; ^4^Dermatology Unit, “Andrea Tortora” Hospital, Azienda Sanitaria Locale Salerno, Pagani, Italy; ^5^Hematology Unit, “Andrea Tortora” Hospital, Azienda Sanitaria Locale Salerno, Pagani, Italy; ^6^Department of Medicine and Health Sciences “Vincenzo Tiberio,” University of Molise, Campobasso, Italy

**Keywords:** Merkel cell carcinoma, ruxolitinib, avelumab, toxicity, management

## Abstract

**Background:** Merkel cell carcinoma (MCC) is a rare neuroendocrine skin cancer. It frequently emerges in the presence of immunosuppression states such as myeloproliferative syndrome (MS). MS is treated with ruxolitinib, a selective JAK1 and JAK2 inhibitor. Avelumab, an anti PDL-1 inhibitor, is the standard treatment for MCC. To date it is unknown if avelumab and ruxolitinib have a synergistic or antagonistic effect when used together.

**Methods:** We have identified all patients diagnosed with MCC, treated with avelumab, concomitant ruxolitinib, belonging to Tortora Hospital, Pagani and Santa Maria La Pietà Hospital, Nola, Italy between June 1 2019 and April 1 2020.

**Results:** Among six MCC patients, we have found two patients in treatment with concomitant drugs. Both patients were being treated with ruxolitinib for MS as a standard regimen without suffering any hematological side effects. After starting doses of avelumab, we found thrombocytopenia, leukopenia, and anemia after cycle 1 and cycle 4, respectively, and decided to suspend both treatments. Following the suspension, the hematological values improved allowing us to restart treatment with avelumab without the need to resume ruxolitinib treatment.

**Conclusions:** The combined treatment of ruxolitinib and avelumab demonstrated severe toxicity. Modifying the schedule or reducing the dose of both drugs needs to be studied in order to be able to treat both pathologies.

## Introduction

Merkel cell carcinoma (MCC) is a rare neoplasm affecting Merkel cells that are part of the neuroendocrine system. MCC has a low incidence rate, about 5–7 cases per million in patients every year. It usually occurs after the age of 50 and most often in men. This carcinoma can arise or follow on from an infection by polyoma virus or after prolonged exposure to UV sun rays (1, 2).

MCC can arise in conjunction with immunosuppressive treatment, as in the case of myeloproliferative syndrome (MS). In fact, in the case of this syndrome there is an abnormal and uncontrolled proliferation of stem cells. A widely used drug in these cases is ruxolitinib, a selective JAK1 and JAK2 inhibitor. Ruxolitinib inhibits the transcriptional activators JAK1 and JAK2 through the inhibition of STAT3 phosphorylation which then cannot interact with JAK and activate it in order to start intracellular communication. Stopping intracellular communication is important in preventing the uncontrolled proliferation of stem cells. During ruxolitinib treatment, it is recommended that periodic checks are undertaken to identify any pre-cancerous skin lesions (3, 4).

Avelumab was approved in 2017 by the Food and Drug Administration (FDA) for MCC immunotherapy (5). It is an anti-PD-L1 human monoclonal IgG antibody, and also works by directly stimulating the lysis of cancer cells through the activation of natural killer cells (6).

To date it is unknown if avelumab and ruxolitinib have a synergistic or antagonistic effect when used together.

## Materials and Methods

All patients with MCC belonging to Tortora Hospital, Pagani and Santa Maria La Pietà Hospital, Nola, Italy, being treated with avelumab, had been diagnosed previously with myeloproliferative syndrome, and were receiving ongoing ruxolitinib therapy were included in our analysis.

The data were collected from June 1 2019 to April 1 2020. The Tortora and Santa Maria La Pietà hospitals are referral hospitals with an average of 400 skin cancer patients receiving diagnosis or treatment every year. Among those patients, ~10 are MCC cancer patients.

## Results

Among six patients with MCC, we found two patients in treatment with concomitant ruxolitinib and avelumab. Patients 1 and 2 needed a large team due to their particular situation; in fact we worked synergistically with a wide range of professionals in different disciplines, including hematologists, dermatologists, and radiologists. One hematologist, five oncologists, and one radiologist evaluated patient 1.

**Figure 1:**
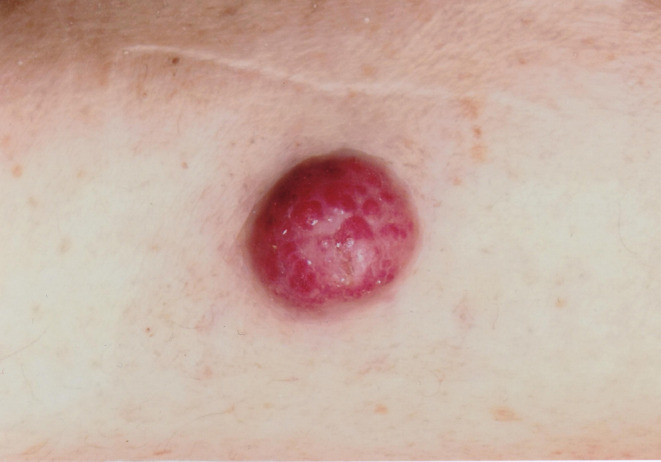
Skin lesion of patient number 1.

The primitive skin lesion of patient 1 is shown in Figure 1. Ga-68 DOTATATE PET/TC was performed on each patient which revealed extensive lymph node metastases. All clinical data are summarized in Table 1.

**Table 1:** Merkel cell carcinoma patient's data.

	**Patient 1**	**Patient 2**
Age	62	73
Sex	F	M
Comorbidity	Hypertension (in treatment with ace inhibitor)	Rheumatoid arthritis
Phototype	Swarthy, dark hair	Clear skin type, red hair
Hematological diagnosis	Polycythemia vera progressed in myelofibrosis in February 2018	Myelofibrosis with splenomegaly from March 2017
Hematological therapy	Ruxolitinib	Ruxolitinib
Beginning of hematological therapy	2018	2017
Diagnosis of MCC	October 2019	February 2019
MCC staging	Inguinal lymph nodes metastases at 68-GA-DOTATATE PET/TC	Inguinal lymph nodes metastases at 68-GA DOTATATE PET/TC
Beginning of therapy for MCC	February 2020	June 2019
Avelumab cycles	6	14
Adverse drug reactions	After cycle I: G4 Thrombocytopenia (Platelet: 19,000); G2 Anemia; G2 Leukopenia	After cycle IV: G3 Thrombocytopenia (Platelet: 45,000); G1 Anemia; G2 Neutropenia
Suspension of ruxolitinib	Yes	Yes

Ruxolitinib did not cause toxicity even if it was administered at the maximum dose indicated by the therapeutic scheme. In fact, both patients had been on treatment for several months with no hematological side effects.

After cycle 1, avelumab patient 1 presented with G4 thrombocytopenia, G2 anemia, and G2 leukopenia, while patient 2 presented with toxicity after cycle 4 with G3 thrombocytopenia, G2 neutropenia, and G1 anemia.

Analyzing the cases, in agreement with the hematologists, radiologists, and dermatologists, it was decided that the treatment of ruxolitinib and avelumab would be suspended in both patients.

Patient 1 after 2 weeks of ruxolitinib suspension had a sharp rise in platelets from 19,000 to 66,000 mm^3^. A month after the suspension, the hematological picture improved with normal levels of platelets, and red and white blood cells.

Patient 2 after 3 weeks of ruxolitinib suspension had normal hematologic values with a platelet count of 140,000 mm^3^.

Following the recovery of the hematological parameters we decided to resume avelumab but to still discontinue the use of ruxolitinib.

In patient 1 avelumab treatment was ongoing while patient 2 had resumed ruxolitinib after the suspension of avelumab for MCC progression disease. Written, informed consent was obtained from the participants for the publication of this case report.

## Discussion

A prior case report has been described by Debureaux et al. showing a synergistic effect from the simultaneous administration of ruxolitinib and nivolumab. This report described high efficacy without toxicity. The authors stated that although the synchronous administration of the two drugs was not indicated, after the third cycle of nivolumab MCC lesions had almost completely disappeared with a good clinical-pathological outcome for the patient (7). It is worth noting that nivolumab, an anti PD1 inhibitor, is effective in MCC as demonstrated from a recent study by Topalian et al. (8), where nivolumab was administered as an adjuvant treatment with a good response.

We describe the first two cases in literature to date with contemporary treatment with ruxolitinib and avelumab.

Since avelumab has a similar action and response in patients to nivolumab it was expected to have the same synergistic effect in the case of concomitant MS (9) as speculated by Debureaux (7).

Instead as shown in our short communication, the result of the concurrent use of the two drugs had the opposite effect.

Instead of being synergistic in our case, the two drugs worked antagonistically. The clinical course of our patients is different from the Debureaux report; in fact we had to suspend the use of ruxolitinib to avoid worsening the patient's health status and to allow for the treatment of MCC.

Ruxolitinib alone, before starting the administration of avelumab, did not lead to thrombocytopenia even if in literature we read that this is one of the potential toxic effects of this drug (10).

Although rare, avelumab has also been seen to reduce red blood cells, white blood cells, and platelets (6).

The biological mechanism of the increased toxicity in this case is unknown; we can only speculate that the risk of hematological toxicity was amplified when the two drugs were used together causing the deterioration in the patients' health.

During this time period, we followed the progression of MS by monitoring the lymphocyte population through flow cytometry in order to check if the absence of ruxolitinib in the treatment caused the hematological prognosis to worsen.

While patient 2 had resumed ruxolitinib treatment after avelumab suspension, for patient 1 we evaluated the re-administration of ruxolitinib and a new possible combination of the two drugs if the myeloproliferative syndrome worsened without needing to stop the administration of avelumab.

We could reintroduce ruxolitinib at a lower dose or reduce both the avelumab and ruxolitinb dosage, or reintroduce ruxolitinib after an objective response for the treatment of MCC.

Close collaboration and multidisciplinary evaluation between hematologists and oncologists is fundamental to this proposition.

## Conclusions

The treatment of avelumab and ruxolitinib demonstrated toxicity when combined. Modifying the schedule, reducing the dose of both drugs or only one, is a necessary to study in order to be able to treat both pathologies.

## Data Availability Statement

The raw data supporting the conclusions of this article will be made available by the authors, without undue reservation.

## Ethics Statement

Written, informed consent was obtained from the participants for the publication of this case reports.

## Author Contributions

GD and LB: study concept, design, and drafting of the manuscript. All authors contributed to the article, acquisition of data, analysis and interpretation of data, and critical revision of the manuscript for important intellectual content.

## Conflict of Interest

The authors declare that the research was conducted in the absence of any commercial or financial relationships that could be construed as a potential conflict of interest.

## Abbreviations

MCC: Merkel cell carcinoma.
